# Expenditure Analysis of HIV Testing and Counseling Services Using the Cascade Framework in Vietnam

**DOI:** 10.1371/journal.pone.0126659

**Published:** 2015-05-15

**Authors:** Van Thu Nguyen, Huyen Thanh Nguyen, Quoc Cuong Nguyen, Phuong Thi Bich Duong, Gary West

**Affiliations:** FHI 360/Vietnam, 7th floor, Hanoi Tourist Building, 18 Ly Thuong Kiet street, Hanoi, Vietnam; UNAIDS, TRINIDAD AND TOBAGO

## Abstract

**Objectives:**

Currently, HIV testing and counseling (HTC) services in Vietnam are primarily funded by international sources. However, international funders are now planning to withdraw their support and the Government of Vietnam (GVN) is seeking to identify domestic funding and generate client fees to continue services. A clear understanding of the cost to sustain current HTC services is becoming increasingly important to facilitate planning that can lead to making HTC and other HIV services more affordable and sustainable in Vietnam. The objectives of this analysis were to provide a snapshot of current program costs to achieve key program outcomes including 1) testing and identifying PLHIV unaware of their HIV status and 2) successfully enrolling HIV (+) clients in care.

**Methods:**

We reviewed expenditure data reported by 34 HTC sites in nine Vietnamese provinces over a one-year period from October 2012 to September 2013. Data on program outcomes were extracted from the HTC database of 42,390 client records. Analysis was carried out from the service providers’ perspective.

**Results:**

The mean expenditure for a single client provided HTC services (testing, receiving results and referral for care/treatment) was US $7.6. The unit expenditure per PLHIV identified through these services varied widely from US $22.8 to $741.5 (median: $131.8). Excluding repeat tests, the range for expenditure to newly diagnose a PLHIV was even wider (from US $30.8 to $1483.0). The mean expenditure for one successfully referred HIV client to care services was US $466.6. Personnel costs contributed most to the total cost.

**Conclusions:**

Our analysis found a wide range of expenditures by site for achieving the same outcomes. Re-designing systems to provide services at the lowest feasible cost is essential to making HIV services more affordable and treatment for prevention programs feasible in Vietnam. The analysis also found that understanding the determinants and reasons for variance in service costs by site is an important enhancement to the cascade of HIV services framework now adapted for and extensively used in Vietnam for planning and evaluation.

## Introduction

Since 1989, when the first report of HIV in Vietnam occurred, the HIV epidemic has been concentrated largely among people who inject drugs (PWID), their sex partners and other key populations at high risk such as female sex workers (FSWs) and men who have sex with men (MSM). Results from the HIV Sentinel Surveillance (HSS) conducted among 41 cities/provinces in Vietnam in 2013 showed that HIV prevalence was 10.3%, 2.6% and 2.3% among PWID, FSWs and MSM, respectively; while only 0.29% among the general population. The country now has more than 80,000 PLHIV receiving ART and there were more than 11,500 newly reported HIV positive cases in 2013 [1–3]. However, the current response to the HIV epidemic has been made possible only through substantial external funding and continued dependence on donors, thus raising major concerns about the sustainability of the service system in Vietnam [4]. Therefore, analyses of expenditure data can foster a better understanding of exactly how financial resources are being expended and can significantly contribute to achieving efficiencies likely to result in more affordable and sustainable services for client and the Government of Vietnam (GVN).

The HIV testing and counseling (HTC) service system is the entry point to the HIV Continuum for Prevention to Care and Treatment (CoPC) services [5]. HTC services help people learn their HIV status and link those found infected to care and treatment. However, in Vietnam, international agencies and donors are currently funding HTC and most other services for people living with HIV (PLHIV). In the 2011–12 fiscal year, 91% of expenditures on HTC were made by international sources [6,7]. Most recently, international donors have begun reducing their financial and managerial support and transition future responsibility for funding services and program management to the GVN. Quantifying the current costs of HTC services will help GVN health officials understand, plan and justify an adequate budget, and mobilize investments and resources during this transition period.

HTC financial and economic costs have been studied in many countries, where costs have ranged from US $6.4 in Uganda [8], US $8.68–19.32 in Swaziland and US $5.05–16.05 in Kenya [9]. In Vietnam, Hoang et al. found that the unit cost per client tested was US $28.40 in 2007 [10]. Though these studies looked at HTC service costs in developing countries, they considered HTC as an independent service with its main purpose helping individuals learn their HIV status. However recent research and evolving programmatic priorities have made it clear that HTC is only one important element of a comprehensive HIV/AIDS services system that has the main goal of engaging and retaining PLHIV in care and treatment services to reduce their viral loads. Reduction of viral loads will extend their lives and reduce risks of further HIV transmission. HTC services must be assessed from this more comprehensive perspective.

The cascade framework, a means of clearly depicting the progress of clients as they enter and move through the CoPC, has been used to assess policies, capabilities and effectiveness of critical functions such as diagnosing HIV, linkages into care, initiation of ART, retention in care, and viral suppression [11, 12]. In Vietnam, analysis of the cascade of HIV diagnosis, treatment and care services in 2012 has shown that of the estimated 248,485 PLHIV, 79.4% were reported through the national reporting system. However, only 29.1% were ever enrolled in care and treatment services [1]. The inadequate linkages between HIV diagnosis and enrollment in care remain a key challenge.

The purpose of this analysis is to estimate the cost to achieve key program outcomes including 1) testing and identifying PLHIV who are unaware of their HIV status, the most important initial step in the CoPC cascade and 2) successfully enrolling HIV (+) clients in care and treatment. Integrating expenditure data into the cascade framework can identify where efficiencies can be achieved in HTC services from identifying HIV positive individuals to successfully referring them to care and treatment services. Additionally, the cost structure was examined to identify the main drivers of the expenses and potential inefficiencies. The results of this analysis will be used to inform the care program and reduce costs to promote sustainability and improve GVN budget planning for future HIV services.

## Methods

### Study setting

The study was conducted in nine provinces (Ho Chi Minh City, Hanoi, Quang Ninh, Hai Phong, Nghe An, Can Tho, An Giang, Dien Bien and Lao Cai) at 34 HTC sites receiving funding through the USAID Sustainable Management of the HIV/AIDS Response and Transition to Technical Assistance (SMART-TA) project (See Table 1). The Provinces currently receiving SMART TA support were purposely selected based on high HIV prevalence among key populations such as people who inject drugs (PWID), female sex workers (FSWs) and men who have sex with men (MSM); HIV prevalence exceeds 20% among PWID in Ha Noi and Quang Ninh, and exceeds 5% among FSWs in Ho Chi Minh City and Hanoi. There is also a burgeoning concentrated epidemic among MSM in Hanoi and Ho Chi Minh City [13].

**Table 1:** The HIV epidemic in the nine provinces with SMART-TA supported HTC sites included in the expenditure analysis.

Province	Region	Population	Current number of PLHIV recorded	Current number of PLHIV estimated	Estimated prevalence of HIV infection (%)
An Giang	South	2,149,407	4,155	6,507	0.32
Can Tho	South	1,188,435	2,612	5,142	0.45
Ha Noi	North	6,779,300	17,093	22,291	0.55
Hai Phong	North	1,878,500	6,896	11,250	0.76
Ho Chi Minh	South	7,485,194		76,002	1.38
Lao Cai	North	648,358	2,365	(*)	(*)
Nghe An	North	1,636,115	4,671	5,851	0.21
Quang Ninh	North	1,144,988	4,530	6,470	0.54
Dien Bien	North	512,268	4,428	(*)	(*)

Source: Vietnam HIV/AIDS Estimates and Projections 2011–2015. Based year: 2012; HIVInfo and program reports, Provincial PACs

(*) Data not available.

Of the 34 HTC sites included in our analysis, 29 are integrated with other HIV Continuum of Prevention and Care (CoPC) facilities, predominantly out-patient clinics (OPCs) and Methadone Maintenance Therapy (MMT) services. The other five sites are stand-alone test sites that refer clients to other CoPC facilities. The service package provided at study sites is nationally standardized with three main elements: (i) a pre-test counseling session; (ii) testing; and (iii) a post-test counseling session, requiring two separate visits, one for testing and one for receiving results.

### Costing Assumptions

The financial cost of HTC services was estimated from the service provider’s perspective and was defined as the monetary value of expenditures for personnel, consumables, and equipment at HTC sites (Table 2). This amount was denoted on invoices and recorded in bookkeeping records as an expense. For staff in integrated sites (i.e. sites providing other services besides HTC), the salary was allocated based on the percentage of working time to provide HTC services. All expenditures incurred in VND were converted into USD equivalents. This analysis did not take into account the expenditures made by other organizations. However, at these sites, the only other known contribution for the HTC program was public land and use of buildings authorized by the GVN. The expenditures currently paid by donors would be the cost that the GVN would assume in the future unless these same services can be provided more efficiently and less costly.

**Table 2:** Components of financial costs for HTC services.

Cost Category	Description
Investment expenditures	Site-level program expenditures, both human and capital, that had a useful life of more than one year
Training (in-service)	Expenditures in this category included expenditures related to training, such as workshop costs, per diem, travel, trainers and facilitators. In-service training supported the further capacity development of existing health workers and program staff *at the site level*.
Equipment and furniture	Site-level expenditures for the purchase of clinical and office equipment and furniture, including computers. Office supplies that were purchased and used up within a year should be recorded as recurrent expenditures.
Recurrent expenditures	Site-level program expenditures that were consumed within the year as part of normal routine program operations
Personnel	All expenditures related to personnel. This included salary, fringe benefits, top-up salary, and reimbursement for overtime.
HIV test kits	Expenditures for the purchase of HIV test kits
Other supplies	Expenditures related to the purchase of any commodities or supplies that are otherwise not classified
Travel and transport	All travel-related expenditures such as airfare, bus fare, per diem, and vehicle fuel and maintenance.
Other recurrent expenditures	Site-level recurrent expenditures otherwise not categorized
Building rental and utilities	Expenses for building rent and utilities (water, electricity, internet, phone…) at sites

### Data collection

Data analyzed covered expenditures from October 2012 to September 2013. Financial data was collected by reviewing the accounting reports submitted by service sites. Shared costs, such as laboratory and administrative costs at integrated OPC, HTC, and MMT sites, were allocated using the direct cost-allocation method [14] based on client volume. Other overhead costs, such as facility management and utility costs, were allocated by personnel and for integrated sites estimates of the HTC portion of expenditures.

Client volume and other characteristics at sites were collected for expenditure allocation among program areas, intervention types/models, and/or target populations. Indicators representing program outcomes were extracted from the program monitoring and evaluation database of 42,390 client records.

### Data processing and analysis

Excel spreadsheets were used to process and analyze the data. Expenses were computed in Vietnamese Dong, and then transferred to U.S $ using the 2013 exchange rate of Viet Nam Dong 20,828 = US $1 [15].

Descriptive statistics were used to examine correlation between actual expenditures and each site’s performance. Unit expenditure per beneficiary was estimated using the following formula:

$$Unit\_Expenditure=\frac{Total\_Expenditure}{HTC\_\mathrm{Pr}ogram\_Indicators}$$

Applying the cascade framework to the HTC program, four program indicators were chosen for analysis including:

- Unit expenditure per client tested for HIV and received results: this indicator directly linked the total expenditure to client volume at each site.
- Unit expenditure per client tested and identified as HIV positive: this indicator was more associated with service utility (use by KPs with high HIV prevalence).
- Unit expenditure per client tested and identified as HIV positive for the first time: this indicator best reflected the “test-and-treat” strategy [11] where applicable.
- Unit expenditure per client tested and identified as HIV positive successfully referred to the OPC: this indicator directly relates to the ultimate goal to enroll or re-engage those identified as HIV positive to care and treatment.

### Ethical consideration

All databases used in this analysis (HTC database and financial reports) did not link to any patient identifying information. The Office of International Research Ethics (OIRE) at FHI 360 reviewed the analysis and determined that it did not meet the regulatory definition of research and/or research involving human subjects as defined under the Department of Health and Human Services Code of Federal Regulations [45 CFR part 46.102(d)(f)] and thus was exempted from the Protection of Human Subjects Committee (PHSC) review.

## Results

### Expenditure and outcomes of HTC services

Among the 34 HTC sites in this analysis, the operational expense for one year ranged from US $2,665 (Quy Chau-Nghe An) to $28,400 (Binh Thanh-Ho Chi Minh City). Expenses for HTC services included expenditures for personnel, HIV test kits, lab services, building rental and utilities, and consumable supplies. HTC programs in rural/remote areas also provided logistical expenses for mobile HTC services and specimen transportation. Other costs included in-service training for HTC staff for further capacity development and service quality improvement and related costs such as per diem and travel expenses. In total, personnel, HIV test kits, and lab services including confirmatory testing comprised the largest proportion of costs, accounting for 40%, 26% and 13% of the total expenses, respectively (Table 3).

**Table 3:** Annual financial costs of providing HTC services by SMART-TA.

Cost categories	Financial cost (US $)	%
*Recurrent Cost*		
Personnel	147,510	40%
HIV test kits	94,666	26%
Lab services including confirmation tests	49,221	13%
Consumable supplies	18,984	5%
Travel/transport	14,741	4%
Building rental & utilities	9,332	3%
*Investment Costs*		
Training (in-service)	20,126	5%
Equipment & furniture	12,938	4%

Table 4 summarizes HTC site characteristics. The mean annual client volume by site was 1500 patients, but varied substantially between sites, ranging from 511 (Bao Thang-Lao Cai) to 2337 in (Ton Duc Thang-Hai Phong) (Table 4). At each HTC site, a counselor explores the HIV risks of clients during pre-test counseling, and then records their KP categories based on self-reported risk. On average, the percentage of HTC clients who were PWIDs, FSWs, MSMs and partners of KPs was 25%, 11%, 3% and 23%, respectively.

**Table 4:** Characteristics of the HTC sites supported by SMART-TA included in the analysis, 2013.

Characteristics	Mean (Range)
Number of individuals that received HTC services and HIV test results	1470 (511–2337)
Number of individuals that received HTC services and HIV (+) test results	95 (14–313)
Number of individuals first ever identified as HIV (+)	64 (9–240)
Number of individuals successfully referred to care and treatment	72 (3–283)

**Fig 1:**
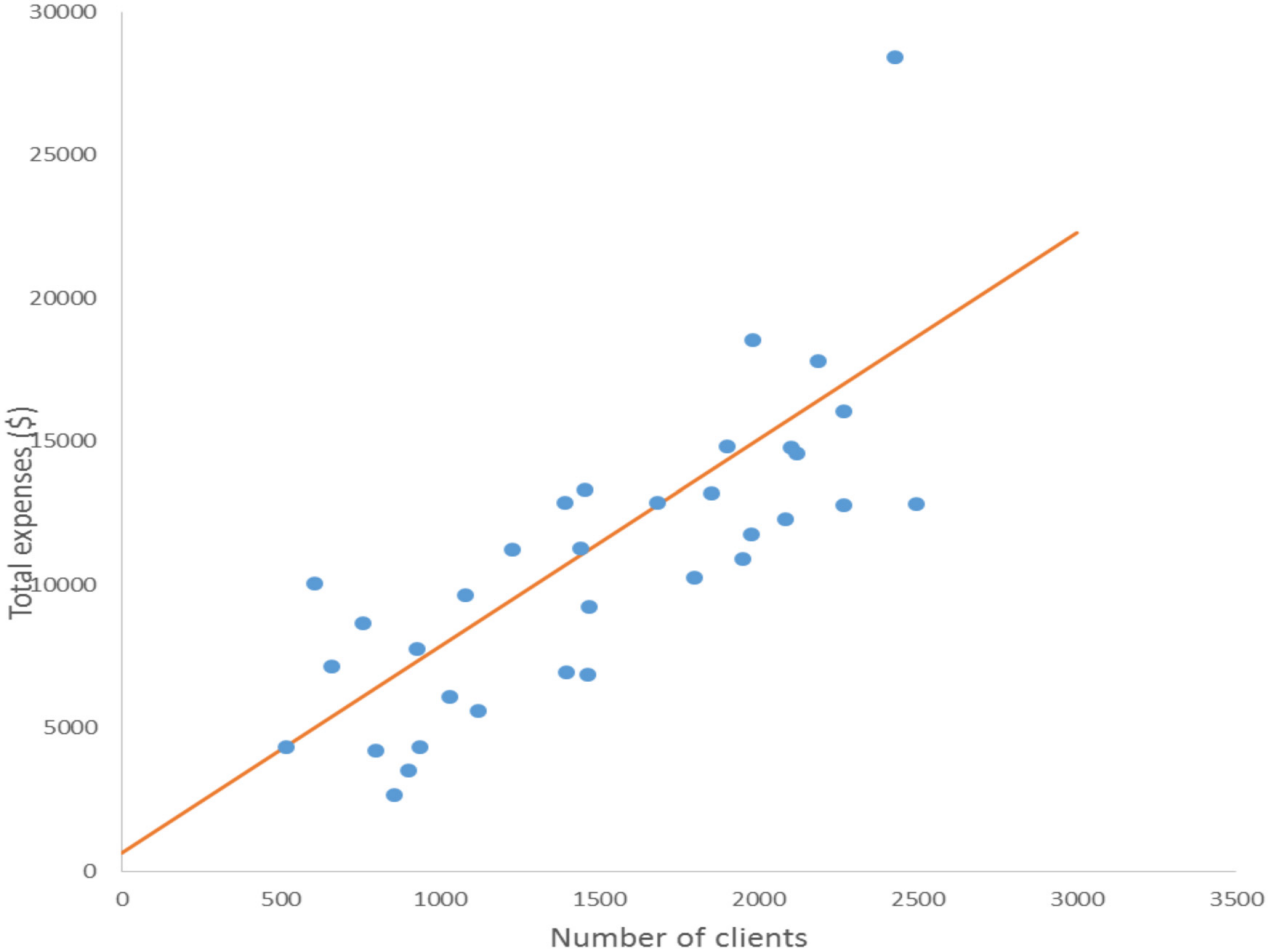
Scatter plot of total expenditure and number of clients for HTC sites supported by SMART-TA.

A scatter plot was used to demonstrate the relationship between total expenditure and number of clients. The R-value of 0.76 suggests a strong positive correlation, the larger client volume was, the higher the expenditure (Fig 1).

### Unit expenditure of HTC

**Fig 2:**
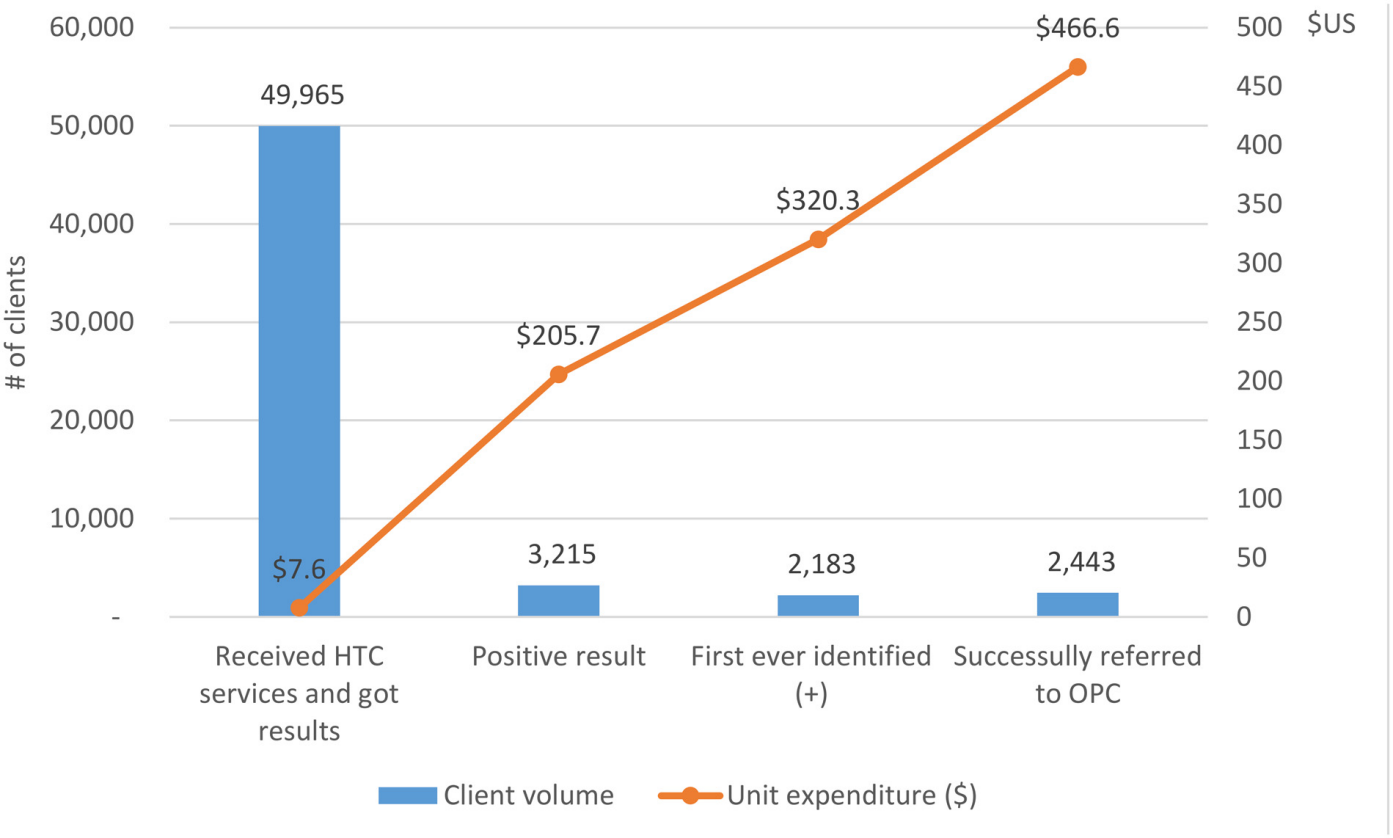
Cascade framework for HTC program and related unit costs.

The mean cost per client tested and received their result was US $7.6, but the unit expenditure per HIV positive was much higher (US $205.7). Adjusted for repeated positive testing, the cost per newly identified HIV positive increased to US $320.3. The mean cost per HIV case successfully referred to care and treatment services was US $466.6 (Fig 2) (Refer to S1 Appendix for site expenditure details).

**Fig 3:**
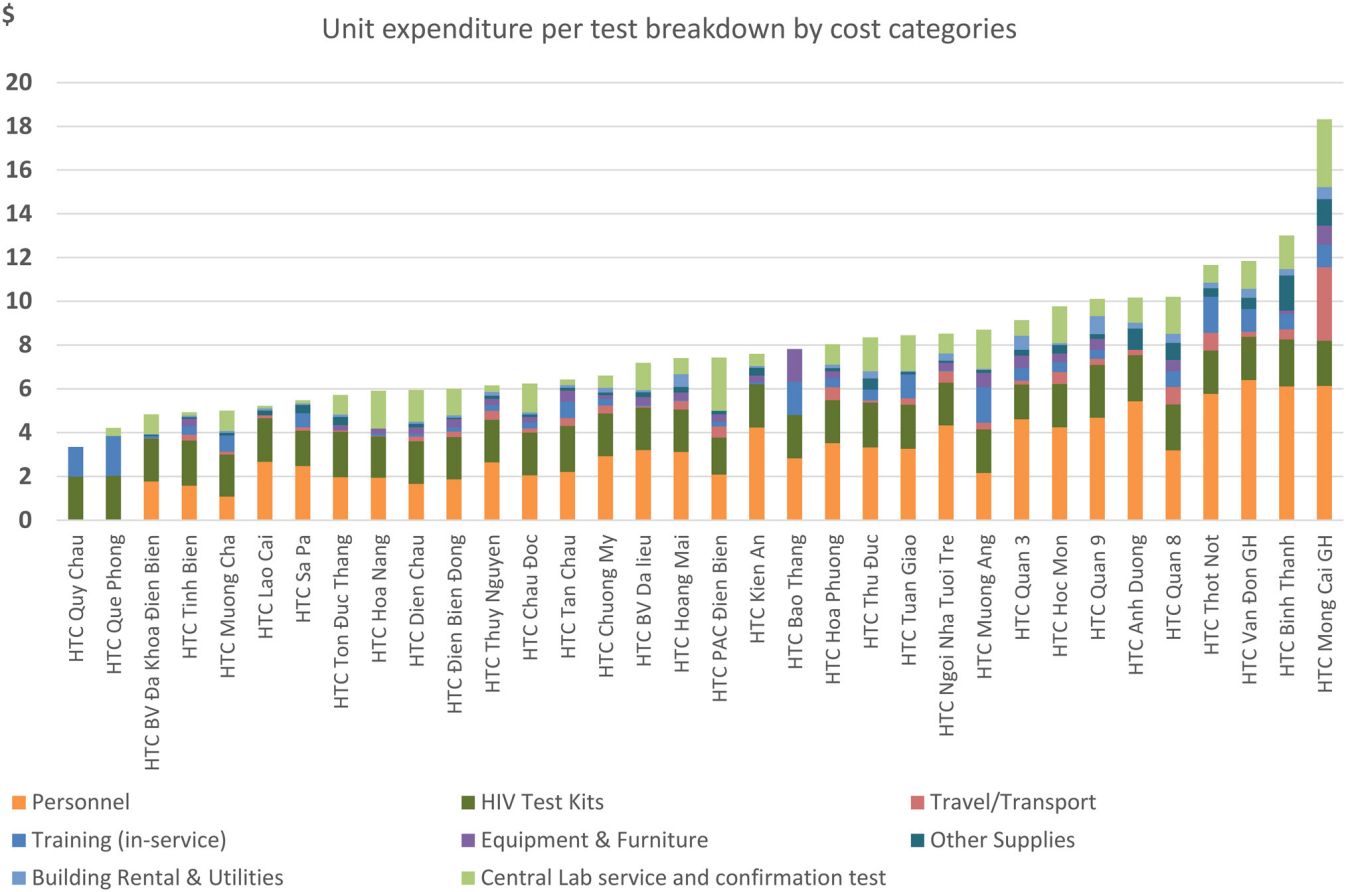
Unit expenditure per HIV test by expense category across HTC sites.

Deeper exploration of the cost for providing HTC services was carried out to identify the main drivers of unit expenditure. Fig 3 shows the unit expenditure per HIV test broken down by expense categories. While the expense for the HIV test kits was similar across sites, the expenditure for human resources per HIV test was very different and was the main factor driving up the unit expense in most sites.

## Discussion

So far published studies appear to have only examined the cost per client tested and cost per HIV (+) positive individual identified. These analyses are too limited as HTC is only one service in a continuum of services (CoPC). But HTC may be the most important service as all other HIV services are dependent on it functioning effectively. By examining the expense data in more detail, and including linkage to care and ART services, our analysis revealed the actual current resources expended for bringing one PLHIV from testing to care and treatment services has a much higher unit cost than diagnosing one HIV (+) client. The reason for this is the weak linkages between HTC and OPC services. Among 3215 clients tested with HIV (+) results, only 2443 (76.0%) were successfully referred to OPC services. In Vietnam, we are currently analyzing the benefits of integrating services and strengthening referral services to improve the linkages and potentially reduce costs for successful referrals.

Our analysis shows both similarities and differences with those from several studies examining the unit cost of HTC services in developing countries. For example, converted to international dollar using base-year of 2012, our unit cost per clients tested (I$19) was similar to findings from a study by Mulogo et al. in Uganda (I$ 16) [8] and Obure et al. in Kenya (I$12.63 – 40.13), and lower than in Swaziland (I$27.13–60.38) [9]. In Vietnam, a study by Hoang et al. [10] collected the financial data of 8 HTC sites in one province in 2007 and found that the unit cost per client tested (I$126.22) was much higher than what is reported in ours (I$19). This difference could be explained by the fact that the volume of HTC services has grown substantially since 2007 when that study was conducted. Since the client volume on average at each site in our sample was much higher than in the Hoang et al.’s study, the lower unit cost may be expected from economies of scale [16].

Our analysis was also able to identify the main drivers for the unit cost of HTC service. While costs for test kits were quite similar across all studied sites (approximately US $2.0 per client tested), expenses for human resources per HIV test varied widely (from US $1.0 to 6.1 per client tested). Over-staffing (i.e. too many management staff serving a moderate number of clients) was identified as the main factor driving up the unit expenditure in many sites. Therefore, reducing the number of staff and task shifting would likely be one viable solution in making services more affordable for the GVN post transition.

Program managers should consider working on a parallel solution to increase the HTC uptake. This could be done by improving the productivity of outreach programs by applying performance-based incentive for peer educators to refer KPs to HTC services. HTC is a gateway for KPs to reach HIV treatment, care, and the full range of prevention options. KPs in Vietnam can access HTC services widely, as they are offered at both health care facilities and stand-alone HTC sites. By the end of 2013, there have been 485 HTC sites established and providing services across Vietnam [13]. However, there remain an estimated 51,130 PLHIVs not being served by the VN CoPC and it is projected that Vietnam will have more than 90,000 new HIV cases from 2013 to 2020 [17]. Thus, there are an estimated total of 141,135 PLHIV that will need services by 2020 and it will not be affordable to the country unless costs are constrained and efficiency is as high as possible. As mentioned earlier, the unit expenditure per PLHIV identified was US$205.7 in 2013. Using a discount rate of 3% [18] and assuming that the number of PLHIV that get tested each year remains the same, the cost of HTC services that the GVN will need to manage will be US $ 32,269,698 by 2020. On average, the cost for the GVN will be US $ 4,033,712 each year just for HTC services. In order to fully finance HTC services when the financial support from international donors ceases, the GVN should adjust potentially expendable costs and mobilize different resources to cover the shortfall.

## Conclusions

The cascade framework has provided meaningful service benchmarks and milestones for tracking clients as they move through the service system. It is feasible to track the costs to reach and diagnose PLHIV and the resources needed to bring them to care and treatment. This analysis identified the main determinants of high costs as too many personnel at site level and low productivity. The findings suggest sites should review their staffing needs and referral systems to ensure there are reasonable workloads and effective, efficient systems in place. Reducing staff costs and improving service performance will be critical to achieving a sustainable service model and affordable costs in Vietnam. We also estimated the cost that the GVN must support and manage in the future, after international donors withdraw their resources. The findings from this analysis can serve as a basis for policy makers and planners in Vietnam to plan, adequately budget, and mobilize resources to sustain critically needed services in Vietnam.

## Supporting Information

S1 Appendix Unit expenditure of the Continuum from Prevention to Care at sites ($). (DOCX)

S1 Dataset (DTA)
