# Supplementary material for: Expenditure Analysis of HIV Testing and Counseling Services Using the Cascade Framework in Vietnam
Source: PLoS One. 2015 May 15;10(5):e0126659. doi: 10.1371/journal.pone.0126659 (PMC4433109; doi:10.1371/journal.pone.0126659)
Supplement: S1 Appendix — (DOCX) [file pone.0126659.s001.docx]

**APPENDIX: Unit expenditure of the Continuum from Prevention to Care at sites ($)**

| SITE | PROVINCE | TYPE  (integrated or stand-alone) | Unit expenditure per HIV test ($) | Unit expenditure per case identified (+) ($) | Unit expenditure per case first ever identified (+) ($) | Unit expenditure per successful referral ($) |
| --- | --- | --- | --- | --- | --- | --- |
| **HTC Chau Doc** | An Giang | I | 6.0 | 154.9 | 346.3 | 170.7 |
| **HTC Tan Chau** | An Giang | I | 6.2 | 59.1 | 76.1 | 92.3 |
| **HTC Tinh Bien** | An Giang | I | 4.7 | 72.2 | 140.0 | 86.8 |
| **HTC Thot Not** | Can Tho | I | 11.2 | 105.3 | 183.7 | 162.8 |
| **HTC Chuong My** | Ha Noi | I | 6.3 | 577.6 | 577.6 | 2,310.4 |
| **HTC BV Da Lieu** | Ha Noi | S | 6.9 | 729.9 | 729.9 | 1,042.7 |
| **HTC Hoang Mai** | Ha Noi | I | 7.1 | 186.4 | 534.3 | 235.7 |
| **HTC Ngoi nha Tuoi tre** | Ha Noi | S | 8.2 | 741.5 | 1483.0 | 1,977.4 |
| **HTC Hoa Phuong** | Hai Phong | I | 7.7 | 313.4 | 1427.9 | 1,606.4 |
| **HTC Kien An** | Hai Phong | S | 7.3 | 439.6 | 439.6 | 1,465.2 |
| **HTC Ton Duc Thang** | Hai Phong | I | 5.5 | 70.5 | 83.9 | 194.5 |
| **HTC Thuy Nguyen** | Hai Phong | I | 5.9 | 423.6 | 455.0 | 558.4 |
| **HTC Binh Thanh** | Ho Chi Minh | I | 12.5 | 90.7 | 175.3 | 133.3 |
| **HTC Anh Duong (CWU)** | Ho Chi Minh | I | 9.8 | 135.2 | 617.4 | 264.6 |
| **HTC Quan 3** | Ho Chi Minh | I | 8.8 | 70.6 | 119.5 | 84.5 |
| **HTC Quan 8** | Ho Chi Minh | I | 9.8 | 45.6 | 53.6 | 56.2 |
| **HTC Quan 9** | Ho Chi Minh | I | 9.7 | 62.8 | 93.1 | 66.2 |
| **HTC Hoc Mon** | Ho Chi Minh | I | 9.4 | 59.2 | 90.6 | 62.1 |
| **HTC Thu Duc** | Ho Chi Minh | I | 8.0 | 58.4 | 162.9 | 52.4 |
| **HTC Bao Thang** | Lao Cai | S | 8.5 | 255.2 | 255.2 | 542.4 |
| **HTC Sa Pa** | Lao Cai | I | 5.3 | 300.1 | 300.1 | 1,400.3 |
| **HTC Lao Cai** | Lao Cai | I | 5.0 | 128.4 | 136.0 | 204.0 |
| **HTC Dien Chau** | Nghe An | I | 5.7 | 115.2 | 157.8 | 131.5 |
| **HTC Hoa Nang** | Nghe An | I | 5.7 | 106.4 | 364.6 | 177.3 |
| **HTC Quy Chau** | Nghe An | I | 3.2 | 46.7 | 65.0 | 95.2 |
| **HTC Que Phong** | Nghe An | I | 4.0 | 22.8 | 30.8 | 40.8 |
| **HTC Mong Cai** | Quang Ninh | I | 17.6 | 287.1 | 502.4 | 1,004.8 |
| **HTC Van Don GH** | Quang Ninh | I | 11.4 | 455.0 | 455.0 | 540.4 |
| **HTC Dien Bien Dong** | Dien Bien | I | 5.8 | 128.3 | 128.3 | 213.8 |
| **HTC Muong Ang** | Dien Bien | I | 8.9 | 150.7 | 150.7 | 150.7 |
| **HTC Muong Cha** | Dien Bien | I | 5.0 | 187.1 | 200.4 | 207.8 |
| **HTC PAC Dien Bien** | Dien Bien | S | 7.1 | 189.5 | 91.2 | 88.5 |
| **HTC BV Da khoa Dien Bien** | Dien Bien | I | 4.6 | 84.7 | 119.9 | 113.6 |
| **HTC Tuan Giao** | Dien Bien | I | 8.1 | 140.7 | 144.3 | 331.0 |
